# Thermochemical CO_2_ splitting performance of perovskite coated porous ceramics

**DOI:** 10.1039/d0ra02353a

**Published:** 2020-06-17

**Authors:** Amir Masoud Parvanian, Hamidreza Salimijazi, Mehdi Shabaninejad, Ulrike Troitzsch, Peter Kreider, Wojciech Lipiński, Mohammad Saadatfar

**Affiliations:** Department of Materials Engineering, Isfahan University of Technology Isfahan 84156-83111 Iran a.parvanian@alumni.iut.ac.ir; Department of Applied Mathematics, Research School of Physics and Engineering, The Australian National University Canberra ACT 2601 Australia mohammad.saadatfar@anu.edu.au; Research School of Earth Sciences, The Australian National University Canberra ACT 2601 Australia; Research School of Engineering, The Australian National University Canberra ACT 2601 Australia

## Abstract

In this paper, we investigate the redox performance of perovskite coated porous ceramics with various architectures. For this purpose, reticulated porous ceramics (RPCs) in three different pore sizes (5, 12, 75 ppi) were fabricated to represent a broad range of structures and pore sizes. The perovskite material is based on lanthanum manganite and was synthesized and doped with Ca and Al through the Pechini method. Using a deep coating method, the surface of RPC substrates was modified by a thin-film coating with a thickness of ∼15 μm. We evaluated the CO_2_ conversion performance of the developed materials in a gold-image IR furnace. X-ray micro-computed tomography along with SEM/EDX were utilized in different steps of the work for a thorough study of the bulk and surface features. Results reveal that the intermediate pore size of 12 ppi delivers the maximum perovskite loading with a high degree of coating homogeneity and connectivity while CO_2_ conversion tests showed the highest CO yield for 75 ppi. Our results show that the extreme conditions inside the furnace combined with the flow of gaseous phases cause the RPCs to shrink in length up to 23% resulting in the alteration of the pore phase and elimination of small pores reducing the total specific surface area. Further our results reveal an important mechanism resulting in the inhibition of CO_2_ conversion where the perovskite coating layer migrates into the matrix of the RPC frame.

## Abstract 2

A representative volume of LCMA coated porous SiC showing a maximum of 23% shrinkage when subject to high-temperature CO_2_ conversion redox reactions. This results in significant structural changes including a reduction in specific surface area.

## Graphical abstract

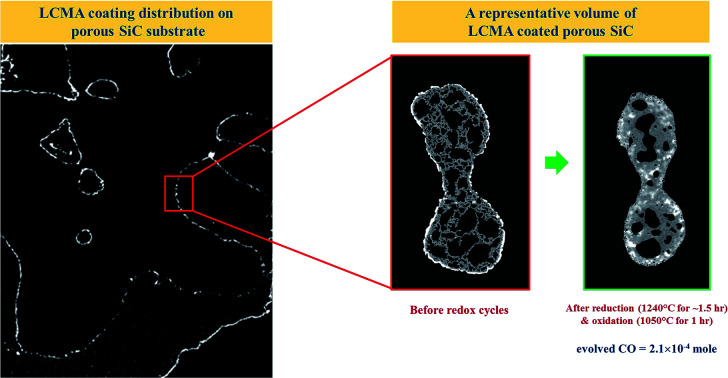

## 1. Introduction

Greenhouse gas effects are understood to be one of the primary causes of climate change. An obvious mitigation strategy is to reduce the emission of harmful gases such as CO_2_. An attractive idea that has gained momentum in recent years is to capture the CO_2_ at the point of emission and convert it into useful products^1–4^ including synthetic fuels, where the entire cycle is powered by renewable resources such as solar irradiation.^5–9^ These solar fuels are very attractive alternatives to non-renewable fossil fuels due to the abundance of solar energy and a near zero emission production cycle. The process of producing solar fuels generally consists of: (i) thermochemical reduction–oxidation (redox) splitting of abundant CO_2_/H_2_O into a CO/H_2_ mixture known as syngas; (ii) hydrocarbon fuel production through some well-established gas to liquid processes (*e.g.* Fischer–Tropsch). However, the commercialization of solar powered syngas has not yet been achieved mainly due to the low solar to fuel efficiency (*η*) of the process.^10^ In recent years, more attention has been paid to the development of redox materials to increase *η* and make the technology commercially viable. The redox materials capability for oxygen exchange (release/uptake of O_2_) directly and proportionally impacts *η* per mass of material.^11^ Oxide materials such as ferrites,^12,13^ hercynite,^14–16^ and more recently ceria^5,8,17,18^ and perovskites,^19–26^ have been successfully used as the redox materials in solar fuel production processes. In particular, perovskites in the form of ABO_3_ (either doped or un-doped on A and B sites) have been the subject of many recent studies even more than ceria due to increased oxygen exchange capacity and lowered reduction temperatures.^20,22–28^ The goal has been to synthesize perovskite crystal structures, with emphasis on the calcium and aluminium doped lanthanum manganite La_1−*x*_Ca_*x*_Mn_1−*y*_Al_*y*_O_3±*δ*_ (LCMA), capable of reaching higher conversion efficiencies. There are two established ways to achieve this goal: (i) creating a larger surface area through which the perovskite can be efficiently exposed to redox reaction,^17,18,23–26,29–32^ (ii) producing unobstructed and homogenous flow of H_2_O/CO_2_ mixture as well as even heat distribution inside the reactor.^18,33–36^ There are two primary steps that can enhance the effect of the redox media in CO_2_/H_2_O splitting process:^11^ (i) effective absorption and transfer of the concentrated solar energy to the reactor and (ii) converting thermal energy into fuel through catalytic reactions using redox materials.^10^ Reticulated porous ceramics (RPCs) are ideal structures that can enhance the conversion efficiency through both of these mechanisms.^18,34–36^

In this paper, we develop a range of perovskite coated RPCs with different pore architecture and geometric characteristics. We then evaluate their mechanical, geometrical and topological properties for syngas production applications. We also develop a robust workflow to coat the RPCs with LCMA perovskite materials and we evaluate the adhesion and quality of coating layers. We utilize a range of techniques including X-ray computed tomography (XCT) as a 3D non-destructive characterization tool, X-ray diffraction (XRD) and high-resolution transmission electron microscopy (HRTEM) to characterize RPC structures and the phase and morphology of perovskite materials. Furthermore, we evaluate the performance of perovskite coated SiC foams by characterizing the CO_2_ conversion efficiency in a high-temperature infrared furnace as a solar-reactor analogue. Finally, we compare the RPC specimens before and after coating as well as post-redox experiments. Using advanced image analysis, we quantify and map spatial and topological changes during material fabrication and experiments.

## 2. Experimental

### 2.1 Fabrication of RPCs with different architecture

Silicon carbide based RPCs were manufactured in different physical properties through polymer sponge impregnation method which is detailed elsewhere.^37^ In this method, a ceramic slurry containing commercial SiC powder (30 μm) and feldspar (65 μm) was prepared and impregnated in a polymer sponge template known as the green product. The RPC substrate was achieved after sintering the green product at 1300 °C. The RPCs were categorized into RPC-A, B and C from coarser to finer pore sizes. Persisting inclusions and debris were removed from the RPC specimens *via* ultra-sonication in ethanol (30 min) following a drying process (100 °C, 30 min).

### 2.2 The synthesis of doped lanthanum manganite and coating on RPCs

**Fig. 1:**
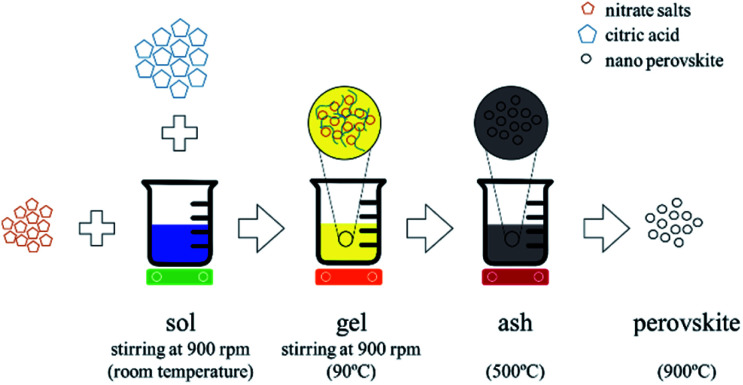
Schematic representation of perovskite synthesis and doping through the Pechini sol–gel based method.

We synthesized calcium and aluminum doped lanthanum manganite (LCMA) with the stoichiometric formula of La_1−*x*_Ca_*x*_Mn_1−*y*_Al_*y*_O_3±*δ*_ (*x* = 0.4, *y* = 0.3) through the Pechini sol–gel based method.^38^ As shown schematically in Fig. 1, the proper weight percentage of the corresponding nitrate precursor materials (*i.e.* La(NO_3_)_3_·6H_2_O, Ca(NO_3_)_2_·4H_2_O, Mn(NO_3_)_2_·4H_2_O and Al(NO_3_)_3_·9H_2_O; all ≥98% purity, Sigma-Aldrich) were dissolved in deionized water and stirred at room temperature to produce the sol followed by the addition of citric acid (C_6_H_8_O_7_ ≥ 99.5% purity, Sigma-Aldrich) at 90 °C as the polymerization and gelation agent. The final product is an ash-like material produced by pyrolysis at 500 °C, which then turned into amorphous perovskite powder through grinding in an agate mortar. A crystallized form of the powder is obtained after heat treatment at 900 °C for one hour (3 °C min^−1^ heating, 5 °C min^−1^ cooling). Subsequently, a coating solution containing 2 g of the perovskite powder and ethanol with a weight ratio of powder : ethanol = 1 : 2 was formed and ultra-sonicated (30 min) to obtain a homogeneous mixture of suspended perovskite powder. The ultra-cleaned RPCs were then dip-coated ten times in the coating solution (dipping speed: 5 mm s^−1^) with the application of pressurized air jet onto the RPCs between each successive dipping. Finally, the perovskite coated RPCs were heat treated (700 °C, 4 h) for extra cohesion strength and stability of the coating on the porous substrate.

### 2.3 XCT imaging of coated RPCs

The X-ray micro computed tomography (XCT) imaging technique was used for 3D scanning of the porous structures. The foam specimens were placed on a motor controlled rotating stage and radioscope projections were taken after each interval of rotation through a micro-focus X-ray source (120 kV) with a resolution of 5 μm per voxel. The 2D projections were processed to reconstruct the full 3D digital foam geometry known as the tomogram with a subsequent image analysis using ANU's Mango software package^39^ to extract valuable information directly from the tomograms. Full 3D structural and topological analyses were performed on samples (i) before and (ii) after perovskite coating, (iii) after redox (CO_2_ conversion) experiments. We evaluated the quality of surface modified porous samples by determining the connectivity of the perovskite coating layer using invariant topological measures.^40,41^ Topological measures such as the Euler characteristic and the Betti numbers of a 3D object can provide insights to the connectivity of the structure. The Euler characteristic, *χ*, is a topological measure for compact bodies, and it is given by the number of components minus the number of cavities. The Betti numbers, *β*_*i*_, count the number of distinct *i*-dimensional cycles, *i.e.*, components *β*_0_, loops *β*_1_, and enclosed voids *β*_2_, in a three-dimensional space. The Euler characteristic is related to the Betti numbers as *χ* = *β*_0_ − *β*_1_ + *β*_2_. Indeed, *χ* is the alternating sum of Betti numbers for 3D objects and it represents an integral geometric measure related to the connectivity of the object.^42,43^ For one sphere *χ* = 2 and for two disjoint spheres *χ* = 4, while a torus (topologically two circles) has *χ* = 0. In other words, every additional hole (loop) contributes by 2 in the Euler characteristic. Smaller values of *χ* and *β*_0_ imply a higher connectivity and a more uniform coating layer. Topological invariants have become an important tool for studying shape in application areas from digital images^44^ to dynamical systems^45^ and disordered structures.^46^

### 2.4 Measuring CO_2_ conversion efficiency of coated RPCs

The redox tests were carried out inside an infrared gold image furnace (Advance Riko Mo. P4C-VHT) where gas flow and temperatures can be accurately controlled, and the effluent gas mixture can be analyzed using a mass spectrometer (PfeifferVacuum Omnistar GSD 320). The reduction and oxidation by CO_2_ were performed at 1240 °C and 1050 °C respectively in line with similar studies reporting substantially higher oxidation in this temperature range.^22^ The LCMA coated RPCs were reduced (at 1240 °C for ∼1.5 hour under 100 sccm argon flow) and oxidized (at 1050 °C for 1 hour in the presence of 10 sccm CO_2_ and 90 sccm Ar).

## 3. Results and discussion

### 3.1 XCT analysis of porous structures

**Fig. 2:**
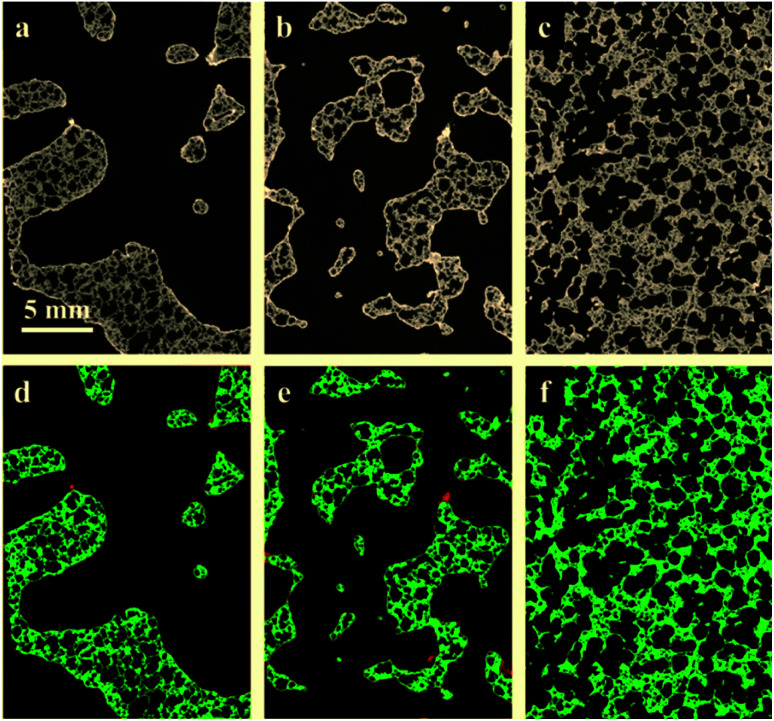
2D slices through the tomograms of the coated RPCs in three different pore architecture from left to right: 5 ppi, 12 ppi and 75 ppi. Top row shows 2D slices through the greyscale tomograms of the samples A (left), B (middle) and C (right). Bottom: the same slices after tomogram segmentation into phases *i.e.* pore (dark), SiC (green) and perovskite coating layer (red).

Fig. 2 presents the tomograms of coated RPCs in three different pore sizes *i.e.* RPC-A, RPC-B, RPC-C before (top) and after (bottom) the separation of phases into pore, solid and perovskite coating phases. Image analysis of the samples reveal an average pore size of 5, 12, 75 ppi (ref. 1) for sample A, B and C, respectively. The volume percentage of each phase is given in Table 1. All the samples have an average of roughly 80% porosity. The solid content where coating adhesion could occur, increases from A to C but coating volume percent is maximum for sample B (2.6 vol%). Pore connectivity analysis shows that the number of closed (isolated) pores increases from sample B (12 ppi) to sample C (75 ppi) in turn reducing the penetration of coating sol. This information is vital when applying the dip-coating process to coat the pore surface of RPCs.

**Table 1:** Statistical parameters extracted from the porous samples' tomograms

Sample	Material phases (vol%)			Pore-coating surface area (mm^2^)	Solid-coating surface area (mm^2^)
	Pore	Solid	Coating		
RPC-A (5 ppi)	84.3	14.6	1.1	621.6	993.2
RPC-B (12 ppi)	80.1	17.3	2.6	1430.2	2238.8
RPC-C (75 ppi)	72.2	26.9	0.9	555.1	1110.9

**Fig. 3:**
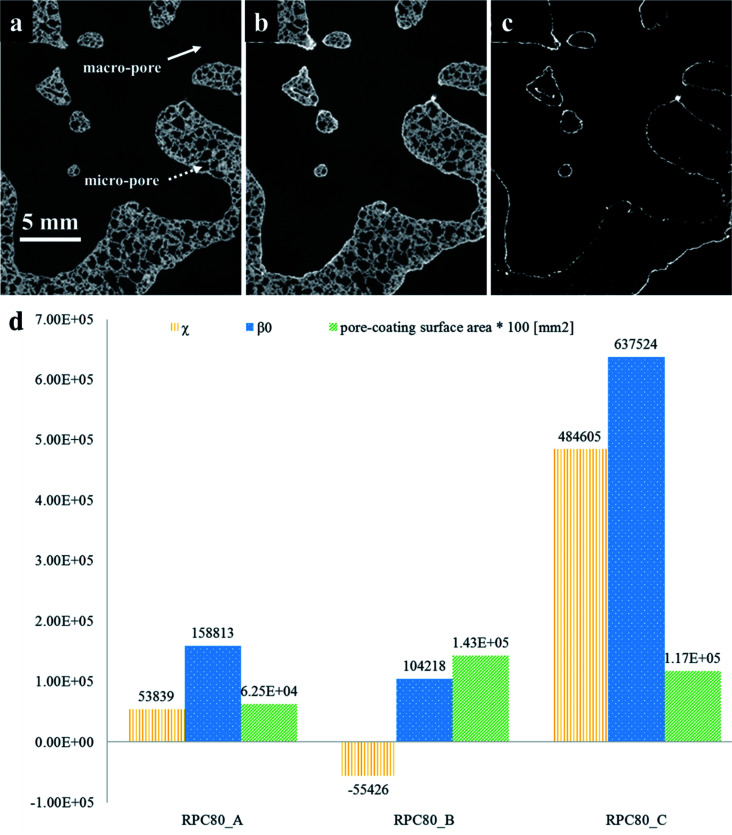
2D slices of sample RPC-B tomograms (a) before; (b) after coating and (c) after registration of (a) and (b); (d) values of topological measures (*χ*, *β*_0_) and the surface area at the interface of pore-coating phases.

The quantification of LCMA layer and detailed inspection of coating quality is possible by comparing the structures before and after coating using 3D image registration techniques. Image registration enables one to geometrically align images of the same object acquired under multiple image modalities or images that are separated by arbitrary lengths of time at different steps of experiment. 3D macro-CT, micro/nano-CT or SEM images can be aligned using similarity-based optimisation techniques.^47^ We register and compare the 3D images of our samples before and after coating, allowing us to quantify changes as a result of coating (Fig. 3). The different pore types *i.e.* macro and micro pores are shown by solid and dashed arrows in Fig. 3(a), respectively. It is clear that the perovskite layer has covered the exterior surface of macro-pores without much penetration into micro-pores. The presence of perovskite coating on the surface of the macro-pores structure can assist with solar irradiation/absorption process *via* the interaction of reactive gases with perovskite material due to the fact that the inactive reaction sites could be minimized. Similar results have been reported recently,^48^ where the active coating sites were selected by impregnating the porous substrates in polymer resins prior to coating. The macro-pores have the potential to provide effective reaction surfaces as well as providing gas mixture conduit in syngas production. Based on the tomograms, we have measured the surface area of the interface between the coating phase and two other phases and the results are summarized in Table 1 along with the total coating specific surface area.

Further, we have computed the connectivity of the perovskite coating layer using the tomograms of the three RPCs. Our calculations are based on two common topological invariants *i.e.* Betti number and Euler characteristic as detailed in Section 2.3.

Fig. 3(d) plots these topological values of porous samples along with the available surface area between the pore and the coating phases. Our results show that sample RPC-B has the most connected and uniform coating (smallest *χ* and *β*_0_ values) followed by RPC-A. Perhaps this is no surprise as the amount of perovskite material present in sample RPC-B is the largest. Indeed, dip-coating process used in this study needs a spongy structure, same as these samples, to allow efficient penetration of the sol through the connected pore network. More interconnected surface area in sample RPC-B with a high SiC matrix vol% led to a more uniform coating. Sample RPC-B also possesses the largest surface area between the coating layer and the pore space providing a higher exposure of the perovskite material.

### 3.2 Elemental mapping and microstructural characterization

**Fig. 4:**
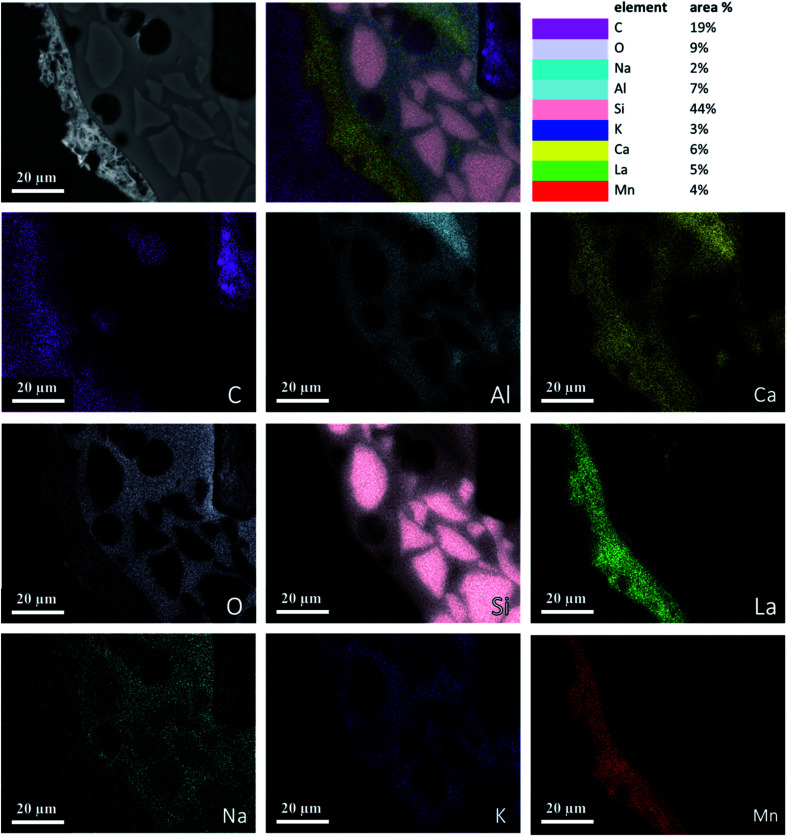
Elemental mapping of a perovskite coated RPC section showing relative proportions of elements.

Fig. 4 is an element map of a cross section of a coated RPC showing the distribution and relative proportion or intensity of different elements. Some elements like Al, Ca, O co-exist in substrate and coating, mainly due to feldspar content of porous substrate, while others such as Si, Na, K are present only in the substrate while La, Mn only exist in the coating layer.

**Fig. 5:**
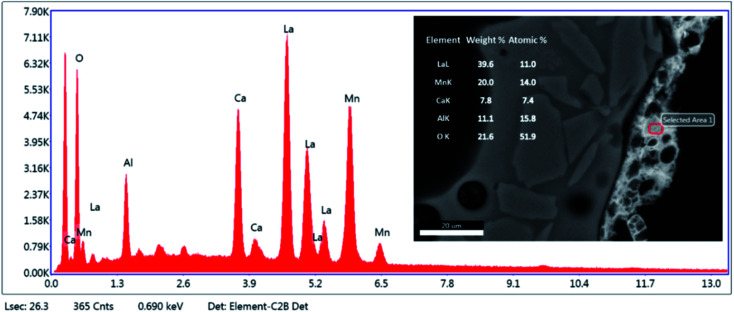
Quantitative EDS analysis of LCMA perovskite coating layer.

Fig. 5 reveals the quantitative EDS analysis of the LCMA coating layer. The heat treatment (at 700 °C) of samples after coating process has led to the diffusion of Al from porous substrate into the coating layer (Fig. 4). The oxygen non-stoichiometry (*δ*) of synthesized LCMA material is measured as 0.4. Non-stoichiometry defines the functionality of perovskite materials through the presence of oxygen vacancies in heterogeneous catalysis processes which mainly enables ionic conductivity in perovskite-based solid solutions.^49,50^ These oxygen vacancies in the crystal structure of perovskite can distort the equilibrium arrangement of other atoms which affects the interactions between neighboring magnetic ions and subsequently induces spin-order transformations.^51^ The oxygen vacancies in perovskite oxides will lead to ionic conductivity characteristics which could be tailored for different functionalities.^52^

### 3.3 CO_2_ conversion of LCMA coated RPCs

**Fig. 6:**
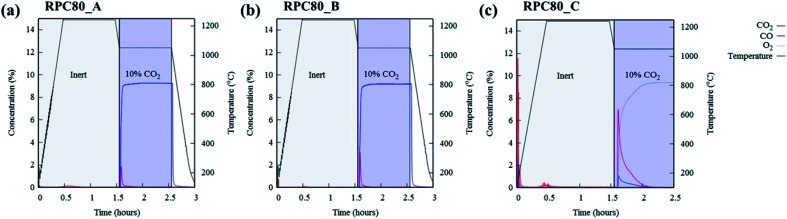
The concentration of reduction/oxidation curves of LCMA coated RPCs for (a) RPC80-A; (b) RPC80-B; (c) RPC80-C.

**Fig. 7:**
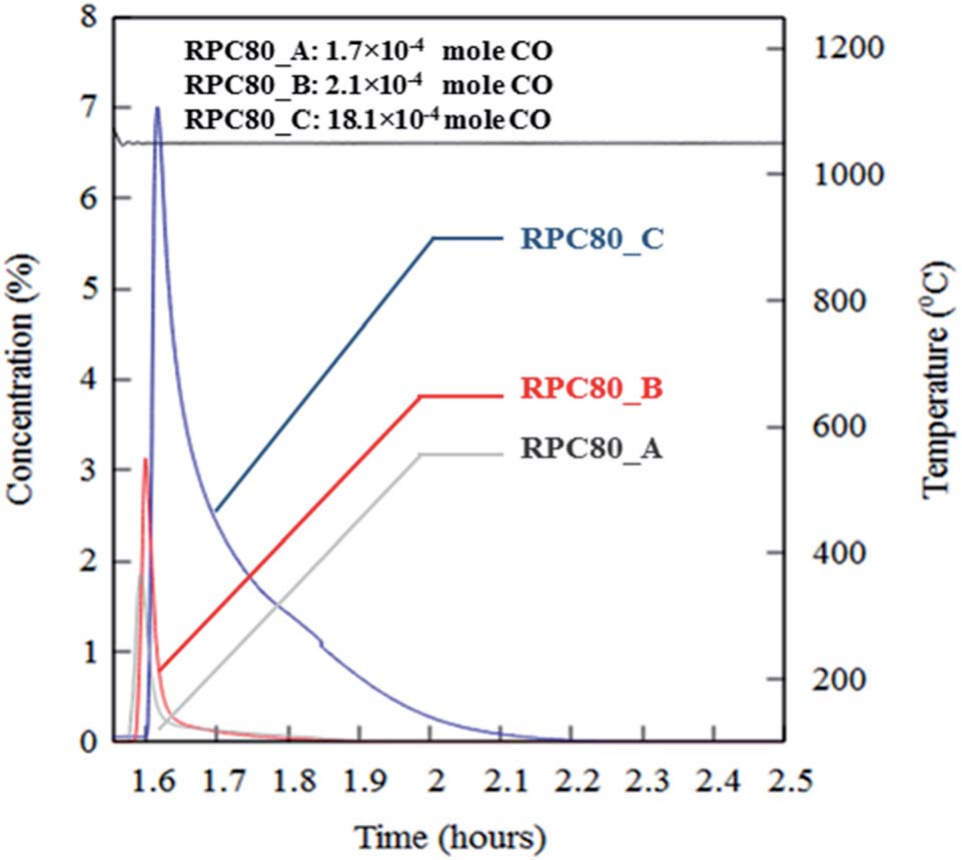
An overlay of oxidation cycles of all coated RPCs with the measured concentration of the evolved CO for a single redox cycle.

Fig. 6(a–c) depicts concentration variations as a function of time for redox cycles of LCMA coated RPCs. The results shown in Fig. 6 imply that after around 1.5 h of reduction at 1240 °C, CO evolved rapidly after exposing the samples to 10 vol% CO_2_ feed gas (at 1050 °C). Fig. 7 illustrates an overlay of oxidation cycles of all samples for a conversion up to ∼40 min with the measured concentration of evolved CO for each sample. These oxidation curves contain an initial sharp peak of CO concentration as a result of rapid oxidation of the material surface followed by slowing down of CO production. The evolved CO mole increases in the order of A (1.7 × 10^−4^ mole), B (2.1 × 10^−4^ mole) and C (18.1 × 10^−4^ mole). Table 2 summarizes coated RPCs' weight measurements including LCMA coating wt% and the amounts of evolved CO per gram of active redox material for each RPC. Since the oxidation reaction rate is highly surface-controlled, we have also provided the specific surface area of coating with pore space for each sample in the last column of Table 2. Our results show that sample RPC-C has the highest amount of evolved CO per gram of perovskite (0.06654 mol g^−1^Table 2). Furthermore, based on the tomographic analysis our data shows that the LCMA coating specific surface area for sample RPC-C is the largest (last column in Table 2). However, this parameter alone does not appear to fully explain the trend of CO yield in samples A and B for the following reason: it increases in the order of B < A < C while the CO conversion increases in the following order A < B < C. Topological analysis of the coating layers (Fig. 3(d)) shows that sample RPC-C has the largest value of *β*_0_ and *χ* among the RPCs and thus possesses the most non-uniform and disjointed spatial distribution of LCMA coating on the surface of porous substrate. We speculate that in addition to the uniformity of the coating layer (*β*_0_ and *χ*) there might be another important factor impacting the conversion efficiency of the coated RPCs. Indeed, tortuosity might be the main defining parameter to explain our results. The highly tortuous flow path within sample RPC-C as a result of smaller interconnected pores could be the main reason for increased interaction between reaction gases and the active surface dramatically enhancing the CO yield. In this case, flow can forcibly touch the active redox material and collaborate into increasing the number of reactions per time unit and thus more CO_2_ conversion efficiency.

**Table 2:** The weight measurement of active redox material on porous samples along with their corresponding evolved CO and LCMA coating specific surface area

Sample code	Weight before coating (g)	Weight after coating (g)	LCMA coating weight (g)	Weight percent of LCMA (wt%)	Evolved CO (mole)	Evolved CO per gram of LCMA (mol g^−1^)	Total specific surface area of LCMA coating (m^−1^)
RPC-A	0.635	0.653	0.018	2.68	1.7 × 10^−4^	0.00971	135.7
RPC-B	0.7	0.721	0.021	2.93	2.1 × 10^−4^	0.00995	114.7
RPC-C	1.093	1.12	0.027	2.43	18.1 × 10^−4^	0.06654	183.1

### 3.4 Post redox structural characterization

**Fig. 8:**
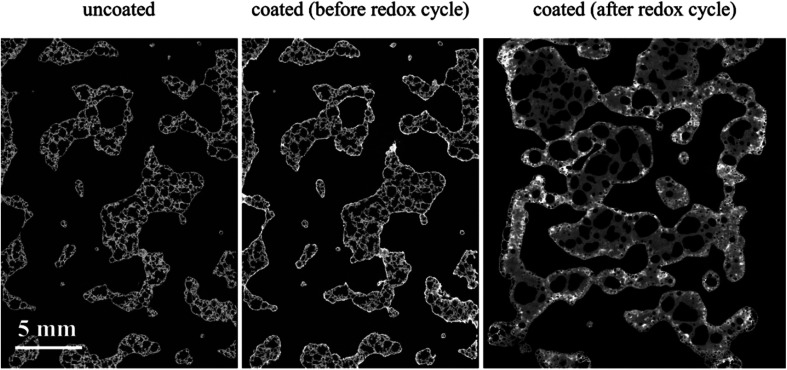
2D slices through the registered tomograms of RPC-B for uncoated (left), coated (middle) and after the redox cycle at reduction: 1240 °C and oxidation: 1050 °C (right).

We have also carried out XCT analysis on specimens after the redox experiments and the results are summarized in Table 3. In this table, the samples' length variation and segmented phase percentages are listed as well as coating specific surface area measured after around 2.5 h of redox cycles. The difference between values before redox experiments is also calculated and presented in parenthesis beside each value. The extreme conditions inside the furnace combined with the flow of gaseous phases caused the LCMA coated RPCs to shrink in length up to 23%. Fig. 8 also compares 2D slices (taken from 3D datasets) of sample RPC-B in uncoated, pre-redox coated and post-redox coated conditions. We observe that the redox cycles causes two effects: (a) the elimination of the macro and micro pores within SiC matrix which caused structural shrinkage and thus an increase in relative phase fraction of both the SiC matrix and LCMA coating; (b) the perovskite material migrates into the SiC matrix which reduces the value of the LCMA coating total specific surface area (Table 3 last column). This is likely governed by the diffusion mechanism as a result of interaction between phases in coating and porous substrate materials at elevated temperatures. There have been other reports in recent studies on the distortion and physical changes in the microstructure and physical properties of the redox material after redox cycles.^53,54^

**Table 3:** The samples' length variation, ave. segmented phase percentages and corresponding LCMA coating specific surface area after around 2.5 h of redox cycles

Sample code	Length variation 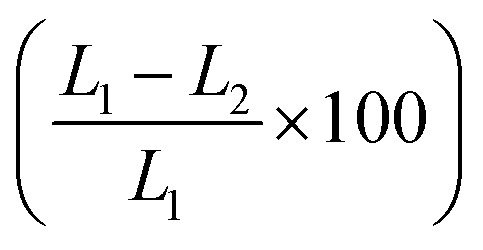 (%)	Ave. segmented phase percentage (vol%)			LCMA coating specific surface area (m^−2^)
		Pore	SiC matrix	LCMA coating	
RPC-A	−10	80.0 (−5)	17.8 (+22)	2.2 (+100)	5.9 (−89)
RPC-B	−23	78.3 (−9)	19.5 (+41)	2.2 (+144)	1.2 (−97)
RPC-C	−15	65.0 (−10)	31 (+15)	4 (+344)	2.5 (−96)

## 4. Conclusions

This paper discusses the redox activity of perovskite coated reticulated porous ceramic (RPC) substrates. SiC based ceramic foams in three different pore architecture and sizes RPC-A (5 ppi), RPC-B (12 ppi) and RPC-C (75 ppi) were manufactured and dip-coated with La_0.6_Ca_0.4_Mn_0.7_Al_0.3_O_3±*δ*_ (LCMA) as the perovskite material. The redox activity of the samples was measured through the conversion of CO_2_ in a gold image furnace. XCT imaging was utilized in different stages of material fabrication and the experiments to detect structural and topological changes in the porous substrates and their active surface. A summary of the results and key findings are presented below:

• Investigated RPCs possess widely different porous architectures despite having pore phase fractions in a narrow range of 72–84% (see Table 1). This foam architecture and pore connectivity affects the dip-coating process where sample RPC-B delivers a balance between pore and solid content with a maximum of perovskite loading 2.6 vol% while this value for RPC-C is 0.9 vol%.

• The effective surface of the RPCs changes due to the addition of perovskite coating layer. A full 3D quantification changes in the effective surface area was calculated by comparing samples' tomograms before and after coating. This revealed a largely homogeneous coating on the surface of macro-pores.

• EDS analysis revealed Al diffusion from the porous substrate into the coating layer as a result of heat treatment. The oxygen non-stoichiometry was calculated to be *δ* = 0.4.

• The connectivity analyses of the coating layer *via* topological measures indicate smallest *χ* and *β*_0_ values of the coating layer for sample RPC-B implying the most connected and uniform coating layer of the three specimens. This sample also possess the largest surface area between the coating layer and the pore space providing a higher exposure of the perovskite materials.

• CO_2_ conversion tests also confirmed an immediate CO evolution when exposed to 10 vol% CO_2_ feed gas. We conclude that the high tortuosity of sample RPC-C as a result of smaller interconnected pores could be the main reason since it increases the interaction between reaction gases and the active surface despite having the most non-homogeneous perovskite coating among the samples.

• The extreme conditions inside the furnace combined with the flow of gaseous phases caused the LCMA coated RPCs to shrink in length up to 23%. This resulted in the elimination of small pores and reduction of pore phase leading to the diffusion of the perovskite material into the SiC matrix. The combined effect was a reduction of the value of the LCMA coating total specific surface area.

## Conflicts of interest

There are no conflicts to declare.

## Supplementary Material
